# Correlation analysis between unenhanced and enhanced CT radiomic features of lung cancers presenting as solid nodules and their efficacy for predicting hilar and mediastinal lymph node metastases

**DOI:** 10.3389/fradi.2022.911179

**Published:** 2022-10-20

**Authors:** Huanchu Yuan, Yujian Zou, Yun Gao, Shihao Zhang, Xiaolin Zheng, Xiaoting You

**Affiliations:** ^1^ Department of Radiology, Dongguan People’s Hospital, Dongguan, China; ^2^ Department of Pathology, Dongguan People’s Hospital, Dongguan, China

**Keywords:** solid nodule lung cancer, tumor staging, radiomics, hilar and mediastinal lymph node metastases, prediction model

## Abstract

**Objectives:**

If hilar and mediastinal lymph node metastases occur in solid nodule lung cancer is critical for tumor staging, which determines the treatment strategy and prognosis of patients. We aimed to develop an effective model to predict hilar and mediastinal lymph node metastases by using texture features of solid nodule lung cancer.

**Methods:**

Two hundred eighteen patients with solid nodules on CT images were analyzed retrospectively. The 3D tumors were delineated using ITK-SNAP software. Radiomics features were extracted from unenhanced and enhanced CT images based on AK software. Correlations between radiomics features of unenhanced and enhanced CT images were analyzed with Spearman rank correlation analysis. According to pathological findings, the patients were divided into no lymph node metastasis group and lymph node metastasis group. All patients were randomly divided into training group and test group at a ratio of 7:3. Valuable features were selected. Multivariate logistic regression was used to build predictive models. Two predictive models were established with unenhanced and enhanced CT images. ROC analysis was used to estimate the predictive efficiency of the models.

**Results:**

A total of 7 categories of features, including 107 features, were extracted. There was a high correlation between the 7 categories of features from unenhanced CT images and enhanced CT images (all *r* > 0.7, *p* < 0.05). Among them, the shape features had the strongest correlation (mean *r* = 0.98). There were 5 features in the enhanced model and the unenhanced model, which had important predicting significance. The AUCs were 0.811 and 0.803, respectively. There was no significant difference in the predictive performance of the two models (DeLong's test, *p* = 0.05).

**Conclusion:**

Our study models achieved higher accuracy for predicting hilar and mediastinal lymph node metastasis of solid nodule lung cancer and have some value in promoting the staging accuracy of lung cancer. Our results show that CT radiomics features have potential to predict hilar and mediastinal lymph node metastases in solid nodular lung cancer. In addition, enhanced and unenhanced CT radiomics models had comparable predictive power in predicting hilar and mediastinal lymph node metastases.

## Background

Lung cancer is the most common malignant tumor in the world; according to statistical data from the Chinese Cancer Society a few years ago, both the morbidity rate and mortality rate of lung cancer rank first (1). Lung cancer staging determines the rationality of the treatment strategy, predicts the prognosis of patients and affects the accuracy of analyzing treatment results (2). Therefore, it is crucial to achieve true staging of lung cancer in the clinic. Computed tomography (CT) scanning is the most important means for diagnosing lung cancer. Long-term clinical observation and study showed that pure ground-glass or subsolid nodules on CT indicate early-stage cancer, including cancer *in situ* or microinvasive carcinoma proven by surgery and histopathology, with generally no lymph node metastasis and a good prognosis by operative treatment (3–5). However, in solid nodule lung cancer, there were large differences in staging in which early-stage cancer without metastasis was propitious to operative treatment and middle- and late-stage lung cancers with hilar and mediastinal lymph node metastases should be comprehensively treated differently (4–6). According to the statistics, up to 8.5% of patients with early-stage cancers that showed solid nodules on CT were found to have lymph node metastasis at pathologic examination. This leads to the deterring of curative effects and a reduction in the survival rate of patients (7). Therefore, the CT staging of solid nodule lung cancer is a clinically difficult point. Other examinations, such as positron emission tomography (PET) and transbronchial ultrasound mediastinal lymph node biopsy, also have some limitations. The price of PET is expensive, and the image resolution is low. Moreover, in transbronchial ultrasound mediastinal lymph node biopsy, an insufficient amount of materials may be collected.

Radiomics is a new technique in which CT, magnetic resonance imaging (MRI) and PET images are inputted to computers, and the internal characteristics of pixel grayscale are extracted by means of specific high-throughput algorithms to unearth a great deal of internal information unidentifiable by the human eye (8). This technique facilitates the deep understanding of the biological characteristics of tumors. At present, radiomics has been highlighted in different disciplines. In a study (7, 9, 10), artificial intelligence and deep learning were applied to predict hilar and mediastinal lymph node metastases of lung cancer, and the predictive accuracy of various models reached up to 0.81–0.82; nevertheless, although the predictive ability was slightly improved, some limitations were inevitable. At present, the rate of inaccurate staging in the clinic reaches 8.5%–10% (11, 12), which does not improve the curative effect. It is unknown whether the above methods are suitable and stable for general clinical applications. In terms of pathological types, non-small-cell lung cancers (NSCLCs) account for a majority of all cases (13), so in our study, solid nodule lung cancer patients meeting the inclusion criteria were used for the radiomic study, and we compared the correlation between unenhanced and enhanced CT imaging, extracted radiomic characteristics and established effective predictive models for hilar and mediastinal lymph node metastases to promote the CT staging accuracy of solid nodule lung cancer in NSCLC.

## Materials and methods

Our hospital review board approved the current study. The requirement for written informed consent was waived for the retrospective cohort.

### Patients

A total of 899 patients with NSCLC confirmed by surgery or histopathology from December 2007 to July 2019 in our hospital were included. The inclusion criteria were as follows: (1) patients had both unenhanced and enhanced CT images, and the slice thickness of the unenhanced and enhanced CT images was no greater than 2 mm; (2) the diameter of solid nodes was greater than 3 mm to allow the precision of segmentation; (3) patients had histopathology and immunohistochemistry results in lung lesions; and (4) the presence or absence of hilar and mediastinal lymph node metastases was confirmed by surgery, aspiration biopsy, or thoracoscopy.

A small number of patients had typical PET manifestations of hilar and mediastinal lymph node metastases (14). Patients with poor image quality and incomplete information were excluded from our study.

A total of 218 patients were finally enrolled, including 109 males and 109 females, ranging in age from 27 to 85 years, with an average age of 59.9 ± 11.1 years (median age, 62 years).

### CT scanning protocol

CT images were acquired using a TOSHIBA Aquilion 4-row spiral CT scanner and a Philips Brilliance 256 iCT scanner. Unenhanced and enhanced examinations were performed. During unenhanced CT scanning, the patients held their breath after deep inspiration. The scanning parameters were as follows: pitch of screw 1, slice thickness and layer distance 1–2 mm, matrix 512 × 512, tube voltage 120 kV, tube current 300 mA, and scanning range from the apex pulmonis to below the diaphragm. In enhanced scanning, a contrast agent (Ultravist solution, 150, 300, 300 mgI/ml) was injected through the hand dorsal vein or ulnar vein at a dose of 1.5 ml/kg of body weight with an injection rate of 3–3.5 ml/s by a power injector (Medrad VCT610, Kanggao Industrial Co., Ltd., USA or YOUWO Bliztwing, Guangzhou Youwo Medical Co., Ltd., China). Imaging of the arterial phase was acquired by a 25-second delay from the beginning of contrast agent injection, and imaging of the venous phase was acquired by a 75-second delay. A filter backprojection algorithm was used for imaging reconstruction. A high-resolution algorithm was used in the lung window, with a window width of 1,250 HU and a window level of 500 HU. A standard algorithm was used in the mediastinal window, with a window width of 250 HU and a window level of 40–60 HU.

### Pathological diagnosis and histopathological verification

Tumor samples obtained from surgery, aspiration biopsy or thoracoscopy were fixed with 10% formaldehyde solution, embedded in paraffin and made into pathological sections, in which the cells were stained with hematoxylin and eosin (HE) and observed under a microscope. For immunohistochemistry, CK, CK7, TTF-1, EGFR, Vimentin, CK5/6, CK14, CK20, Villin, P63, and Ki67 were studied.

### Imaging observations and methods of histopathological verification

Observing and analyzing of CT imaging was performed by two chest radiologists (10 years of experience, advice-senior professional title and senior professional title). The two chest radiologists observed CT manifestations of lesions in lung and hilar andmediastina and recorded data for statistical analysis. Histopathological and immunohistochemical analyses of lung solid nodules were performed in all patients. The 114 patients underwent removal operations of hilar and mediastinal lymph nodes or thoracoscope biopsy to determine whether lymph nodes exhibited abnormalities, and histopathological diagnoses were acquired. Another 5 patients who had no histopathological data enlargement of the hilar and mediastinal lymph nodes number, enhancement of venous phases on CT were observed. Meanwhile clearly high metabolic state on PET was observed.

### Radiomic feature extraction

#### Tumor segmentation

**Figure 1:**
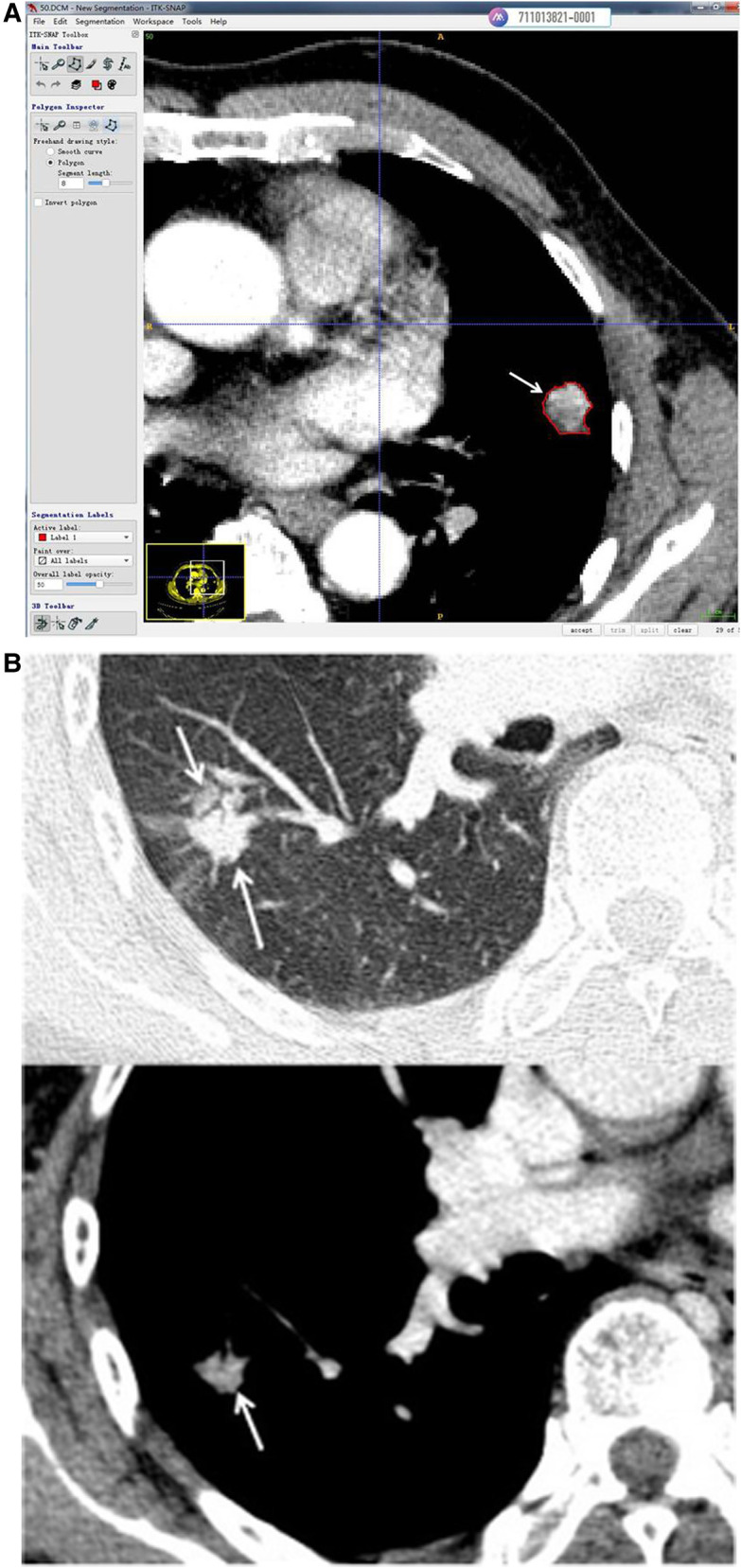
(**A**) Use of the snake-shaped segmentation tool of ITK-SNAP software (version 3.8.1, www.itksnap.org) to trace the contour profile of solid tumors in CT of the mediastinal window. This solid nodule of the left lung was enhanced (venous phase, 2 mm slice thickness) and cut along the tumor margin (arrow). (**B**) This part solid nodule of the right lung was enhanced (venous phase, 2 mm slice thickness). The node presented part ground glass and part solid in lung window on up imaging (arrow). The solid part were displayed in mediastinal window(arrow) and segmentation was done along the margin of solid part (arrow).

The snake-shaped segmentation tool of ITK-SNAP software (version 3.8.1, www.itksnap.org) (15) was used to trace the contour profile of solid tumors on the mediastinal window of the CT images. As shown in Figures 1, 3D segmentation in slice by slice of whole tumors was performed on enhanced and unenhanced images. To part-solid nodules, the solid parts were taken with adjusting of window width and window lever. Venous phase images were adopted in enhanced scanning. Segmentation of nodules were done by two chest radiologists of the above by consulting with each other.

#### Feature extraction

**Figure 2:**
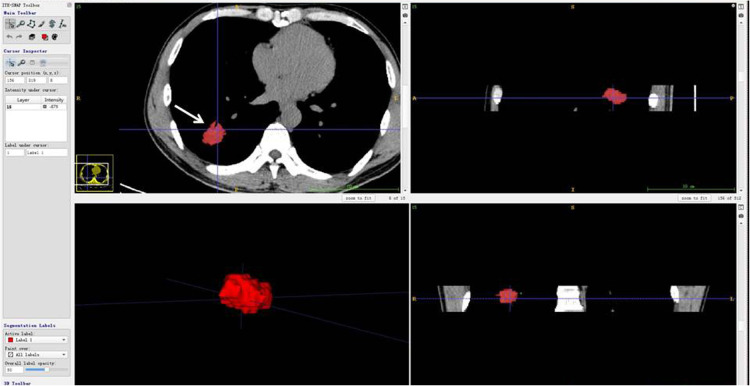
CT scan of the patient (woman, 77 years old) showing a solid nodule in the post-basal segment of the right lower lobe, which proved to be lung infiltrating adenocarcinoma. The 3-dimensional imaging (all slices) of segmentation was imported into AK software (Artificial Intelligence Kit, Version 3.3.0, GE Healthcare) (13) for feature extraction. The imaging presented unenhanced imaging: the left upper figure was cut into images (arrow), the left lower figure was cut into whole nodules, and the upper and lower figures on the right are vertical plane and coronal plane images of the cut nodules.

The CT images and segmentation files were imported into AK software (Artificial Intelligence Kit, Version 3.3.0, GE Healthcare) (16) for feature extraction (Figure 2). A total of 107 features in 7 categories were extracted, including 14 first-order features, 5 shape features, 18 gray level co-occurrence matrix (GLCM) features, 16 gray level run length matrix (GLRLM) features, 16 gray level size zone matrix (GLSZM) features, 24 gray level dependence matrix (GLDM) features and 14 neighboring gray tone difference matrix (NGTDM) features.

The details of the features are listed in Supplementary Appendix Table 1.

#### Feature processing

First, the features with a variance equal to 0 were removed. Then, the *Z* score was used to standardize the radiomics features.

### Data analysis and statistical analysis

Correlation analysis was performed on the features extracted from unenhanced and enhanced CT imaging using Spearman rank correlation analysis. According to the presence of hilar lymph node metastasis, the patients were divided into a lymph node metastasis group and a non-lymph node metastasis group. The patients were randomly divided into training and test groups at a ratio of 7:3 in that numbers of training and test groups were respectively 83 and 36 cases in lymph node metastasis group, ones of training and test groups were respectively 69 and 30 cases in non-lymph node metastasis group. The Mann–Whitney *U* test, Spearman rank correlation analysis and the maximum relevance–minimum redundancy (MRMR) algorithm were used for feature selection in the training group. In the Mann–Whitney *U* test, features that were significantly different between the lymph node metastasis group and the non-lymph node metastasis group were included (*p* < 0.05). Spearman rank correlation analysis was used to evaluate the correlation between features. When the correlation between two features was greater than 0.75, one of them was randomly reserved. Finally, we adopted the MRMR algorithm, and the 5 most valuable features were preserved. Multivariate logistic regression was used to build the predictive models. Two predictive models were constructed based on enhanced and unenhanced CT features. The area under the curve (AUC), accuracy, sensitivity and specificity were calculated to estimate the predictive efficiency of the predictive models. DeLong's test was used to compare the AUCs of the two models.

For clinical information, categorical variables are expressed as frequencies (percentages) and were compared between groups using the chi-square test. Continuous variables are expressed as the medians (interquartile ranges) and were compared between groups using the Mann–Whitney *U* test. All statistical analyses in the present study were performed with R (version 3.6.0). A two-tailed *p* value <0.05 indicated statistical significance.

## Results

### General CT morphological characteristics, pathological types and groups of lung cancer nodules

There were 218 nodules in 218 patients, and the tumors were located in various lobes of both lungs.

**Figure 3:**
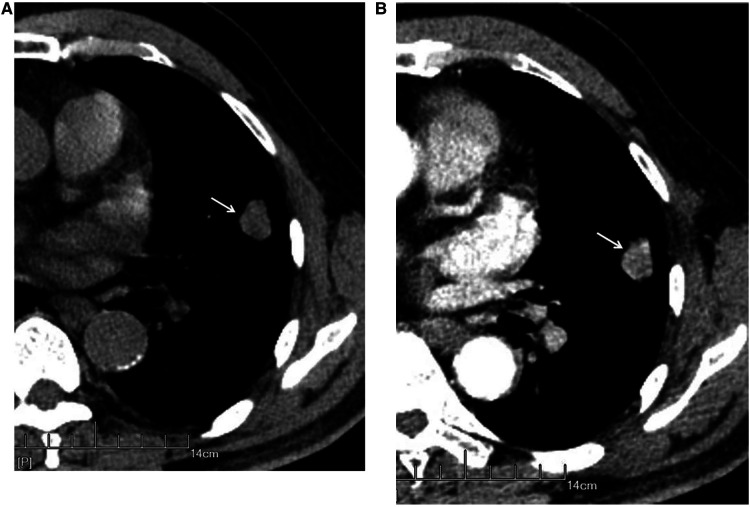
Male, 69 years old, with infiltrating adenocarcinoma in the anterior segment of the upper left lung (moderately differentiated degree) without hilar and mediastinal lymph node metastases. CT showed a solid nodule. The left figure (**A**) shows unenhanced imaging and a solid nodule (arrow), and the right figure (**B**) shows enhanced imaging and an enhanced nodule (arrow).

**Figure 4:**
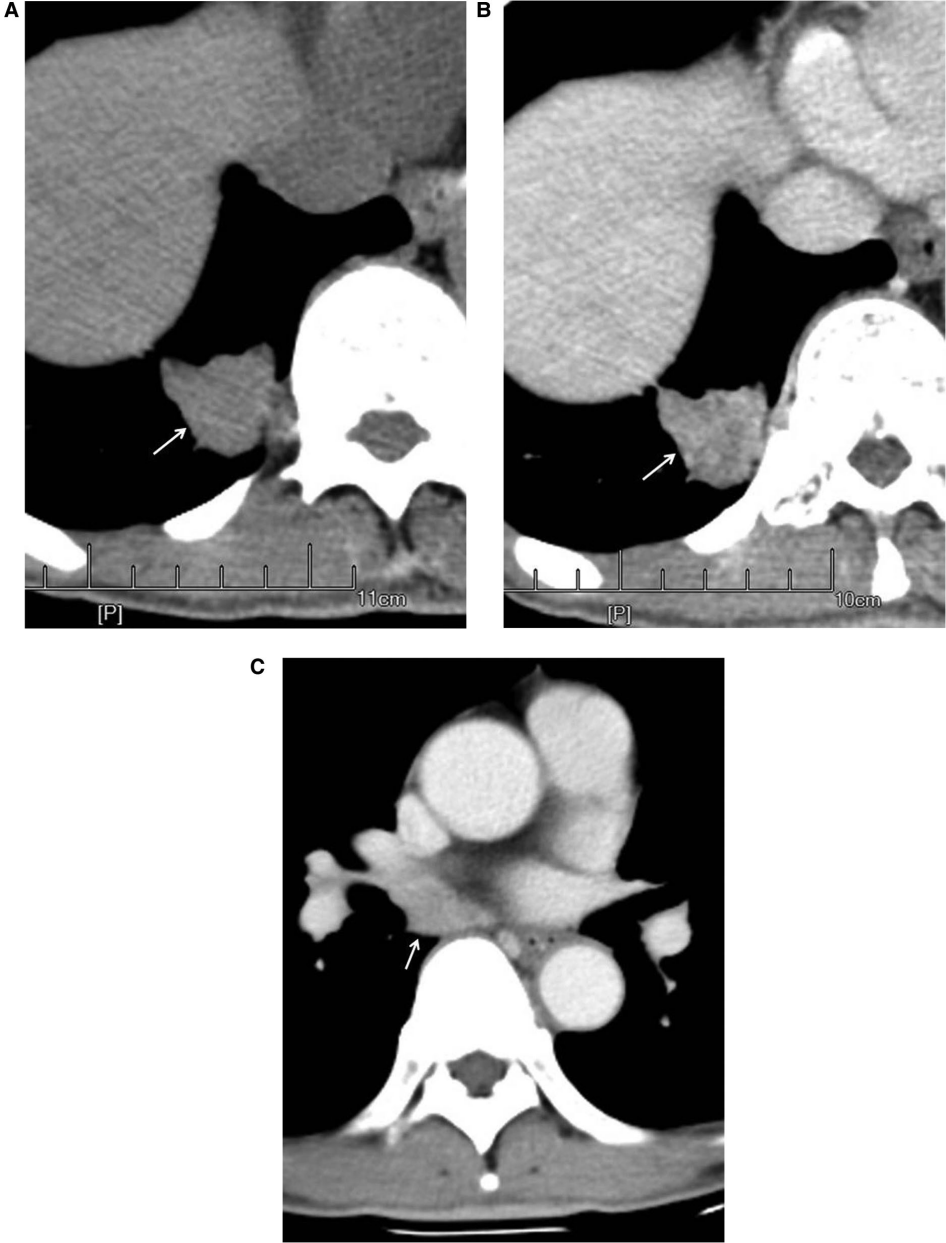
Male, 67 years old, with adenosquamous carcinoma in the medial basal segment of the lower right lung (moderately differentiated degree) with hilar and mediastinal lymph node metastases. CT showed a solid nodule. The left figure (**A**) shows unenhanced imaging and a solid nodule (arrow). The right figure (**B**) shows enhanced imaging and a heterogeneously enhanced nodule (arrow). Figure (**C**) shows lymphadenectasis in the right lung hilum and subtuberances (arrow) that were lymphatic metastases proven by pathology.

The nodules were mainly composed of soft tissue, and some nodules contained ground-glass components and calcium zones. For the pathological types, there were 212 cases of adenocarcinoma (including 3 cases of colloid carcinoma), 1 case of squamous cell carcinoma, and 5 cases of adenosquamous carcinoma. There were 119 patients with no hilar or mediastinal lymph node metastasis (Figure 3) and 99 patients with hilar and mediastinal lymph node metastases, which manifested as ipsilateral hilar and mediastinal lymph node enlargement (Figure 4). The basic clinical information, CT manifestations and pathological types are shown in Table 1.

**Table 1:** Detailed clinical information, CT manifestations and pathological types.

Item	Data	Item	Data
		Location	
Sex			
Male	109 (50.00%)	Right lobe	
Female	109 (50.00%)	Superior lobe	80 (36.70%)
Age			
Total	62 [53.00, 68.00]	Middle lobe	16 (7.30%)
Male	62 [55.00, 69.00]	Apical segment of inferior lobe	16 (7.30%)
Female	61 [51.00, 66.00]	Basal segment of inferior lobe	23 (10.60%)
Tumor size (mm)			
Minimum dimension	20 [15.00, 25.00]	Left lobe	
Maximum dimension	25 [19.30, 33.00]	Superior lobe	48 (22.00%)
Density		Lingula lobe	4 (1.80%)
Solid	151 (69.30%)	Apical segment of inferior lobe	8 (3.70%)
Part solid	67 (30.70%)	Basal segment of inferior lobe	23 (10.60%)
Calcification			
Yes	20 (9.20%)		
No	198 (90.80%)		
Pathological type		Surgery/biopsy	
Adenocarcinoma	209 (95.90%)	Surgery	176 (80.70%)
Mucinous adenocarcinoma	3 (1.40%)	Biopsy	37 (36.70%)
Squamous cell carcinoma	1 (0.40%)	PET-CT	5 (2.30%)
Adenosquamous carcinoma	5 (2.30%)		

### Correlation analysis of radiomic features between unenhanced and enhanced CT images

**Figure 5:**
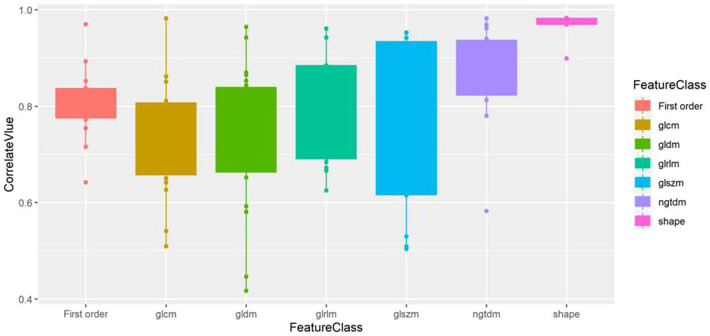
Box plot of the correlation value of various types of features between unenhanced and enhanced images. Shape features exhibit the highest correlation.

The correlation analysis results of the 7 types of features are displayed in Table 2 and Figure 5, which show that the features from unenhanced images have a strong correlation with the features from enhanced images. Among them, the shape features had the strongest correlation, the correlation coefficient [median (interquartile range)] was 0.98 (0.97, 0.98), and the value range was 0.90–0.98. The median correlation coefficients of NGTDM, GLRLM and first-order features between unenhanced and enhanced images were all greater than 0.8. The median correlation coefficients of GLCM, GLSZM and GLDM between unenhanced and enhanced images were all greater than 0.7.

**Table 2:** Correlation of various features in unenhanced and enhanced imaging.

Feature type	Correlation coefficient [median (interquartile range)]	Value range
First order	0.8 [0.78,0.84]	0.64–0.97
GLCM	0.75 [0.66,0.81]	0.51–0.98
GLDM	0.73 [0.66,0.84]	0.42–0.96
GLRLM	0.84 [0.69,0.88]	0.62–0.96
GLSZM	0.74 [0.62,0.93]	0.5–0.95
NGTDM	0.9 [0.82,0.94]	0.58–0.98
Shape	0.98 [0.97,0.98]*	0.9–0.98

* The correlation coefficient of shape was the highest.

### Predictive models for predicting hilar and mediastinal lymph node metastases

The detailed clinical data distributions of the patients in the training and test groups are displayed in Table 3. There were no significant differences in any of the variables between the training group and the test group (all *p* > 0.05).

**Table 3:** Clinical data of patients in the training group and test group.

Variable	Training group	Test group	*p* value
Age	61.00 (51.00, 67.55)	62.00 (56.95, 68.00)	0.393
Tumor size (mm)			
Short	19.50 (15.00, 28.00)	20.00 (14.95, 24.00)	0.605
Long	24.00 (19.00, 34.00)	26.00 (20.00, 31.00)	0.975
Sex			
Male	74 (48.68%)	35 (53.03%)	0.555
Female	78 (51.32%)	31 (46.97%)	
Density			
Solid	45 (29.61%)	22 (33.33%)	0.584
Part solid	107 (70.39%)	44 (66.67%)	
Calcification			
Yes	138 (90.79%)	60 (90.91%)	0.978
No	14 (9.21%)	6 (9.09%)	
Location			
Right lobe	55 (36.18%)	25 (37.88%)	0.481
Superior lobe	16 (10.53%)	7 (10.61%)	
Middle lobe	11 (7.24%)	5 (7.58%)	
Apical segment of inferior lobe	10 (6.58%)	6 (9.09%)	
Superior lobe	38 (25.00%)	10 (15.15%)	
Ligule lobe	2 (1.32%)	2 (3.03%)	
Apical segment of inferior lobe	7 (4.61%)	1 (1.52%)	
Basal segment of inferior lobe	13 (8.55%)	10 (15.15%)	
Pathological type			
Adenocarcinoma	145 (95.39%)	64 (96.97%)	1.0
Mucinous adenocarcinoma	2 (1.32%)	1 (1.52%)	
Squamous cell carcinoma		0 (0.00%)	
Adenosquamous carcinoma	4 (2.63%)	1 (1.52%)	
Verification method			
Surgery	120 (78.95%)	56 (84.85%)	0.657
Biopsy	28 (18.42%)	9 (13.64%)	
PET-CT	4 (2.63%)	1 (1.52%)	

In the unenhanced model, 91, 20 and 5 features remained after the Mann–Whitney *U* test, Spearman rank correlation analysis and MRMR algorithm, respectively. In the enhanced model, 93, 17 and 5 features remained after the Mann–Whitney *U* test, Spearman rank correlation analysis and MRMR algorithm, respectively. Table 4 presents the coefficients of the remaining features in the multivariate logistic regression. The radiomic scores of the two predictive models were constructed and are as follows:

**Table 4:** Coefficient of features in the unenhanced and enhanced models.

Feature name	Coefficient
Unenhanced	
(Intercept)	−0.562625
glrlm_GrayLevelNonUniformity	0.196269
ngtdm_Strength	−2.72152
glszm_HighGrayLevelZoneEmphasis	0.415081
glrlm_GrayLevelNonUniformityNormalized	0.644457
glrlm_RunEntropy	0.37077
Enhanced	
(Intercept)	−0.503774611
gldm_DependenceVariance	0.343004274
glszm_SmallAreaLowGrayLevelEmphasis	−0.489234723
glszm_SmallAreaEmphasis	0.581393866
firstorder_TotalEnergy	0.427128583
gldm_DependenceEntropy	1.373260326

Formula of unenhanced prediction model:

$$\begin{array}{rl} \mathrm{Radscore} & \_unenhanced\;=\;U\_\mathrm{original}\_\mathrm{glrlm}\_\mathrm{GrayLevel}\\ & \mathrm{NonUniformity}*(0.196)\\ & +U\_\mathrm{original}\_\mathrm{ngtdm}\_\mathrm{Strength}*(\text{-}2.722)\\ & +U\_\mathrm{original}\_\mathrm{glszm}\_\mathrm{HighGrayLevelZone}\\ & \;\;\mathrm{Emphasis}*(0.415)\\ & +U\_\mathrm{original}\_\mathrm{glrlm}\_\mathrm{GrayLevelNonUniformity}\\ & \;\;\mathrm{Normalized}*(0.644)\\ & +U\_\mathrm{original}\_\mathrm{glrlm}\_\mathrm{RunEntropy}*(0.371)+(\text{-}0.563). \end{array}$$

Formula of enhanced prediction model:

$$\begin{array}{rl} \mathrm{Radscore} & {}_{e}nhanced\;=\;E\_\mathrm{original}\_\mathrm{gldm}\_\mathrm{Dependence}\\ & \mathrm{Variance}*(0.343)\\ & +E\_\mathrm{original}\_\mathrm{glszm}\_\mathrm{SmallAreaLowGrayLevel}\\ & \;\;\mathrm{Emphasis}*(\text{-}0.489)\\ & +E\_\mathrm{original}\_\mathrm{glszm}\_\mathrm{SmallAreaEmphasis}*(0.581)\\ & +E\_\mathrm{original}\_\mathrm{firstorder}\_\mathrm{TotalEnergy}*(0.427)\\ & +E\_\mathrm{original}\_\mathrm{gldm}\_\mathrm{DependenceEntropy}*(1.373)\\ & +(\text{-}0.504) \end{array}$$

### Prediction efficiency of unenhanced and enhanced models for hilar and mediastinal lymph node metastases

**Figure 6:**
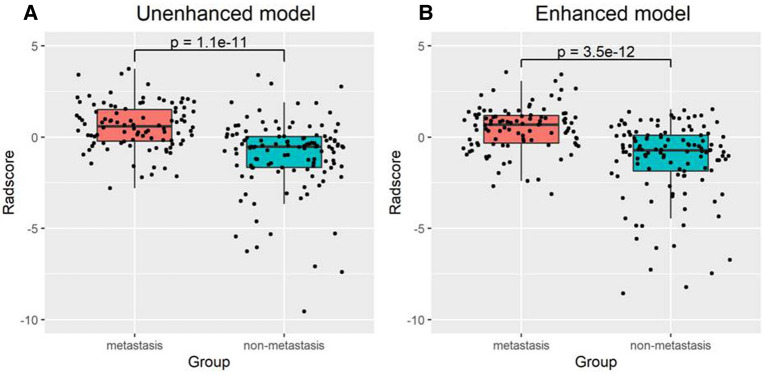
Box plot of the Radscore value in the unenhanced (A) and enhanced (B) models.

**Figure 7:**
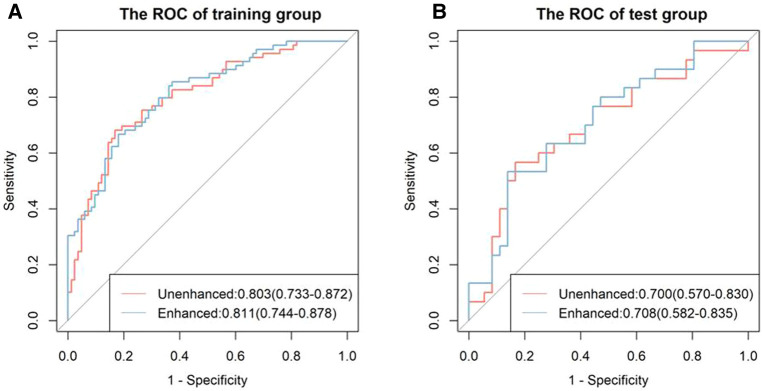
ROC curves of the unenhanced and enhanced models. The left figure (A) shows the ROC curve of the training cohort, and the right figure (B) shows the ROC curve of the test cohort. There was no significant difference between the two models in the training and test groups (both *p* < 0.05).

There was a significant difference in the radiomic score of the unenhanced and enhanced models between the lymph node metastasis group and the non-lymph node metastasis group. Figure 6 shows the boxplot of the radiomic score of the unenhanced and enhanced models. The AUCs of the unenhanced model for predicting hilar and mediastinal lymph node metastases were 0.803 and 0.700 in the training and test sets, respectively. The AUCs of the enhanced model for predicting hilar and mediastinal lymph node metastases were 0.811 and 0.708 in the training and test sets, respectively. The specificity and sensitivity of the two predictive models are shown in Table 5, and the ROC curve is presented in Figure 7. There was no significant difference in the prediction efficiency of the two prediction models (DeLong's test, *p* = 0.788 for the training set and *p* = 0.885 for the test set).

**Table 5:** Prediction efficiencies of the unenhanced and enhanced models.

CT scanning	Group	AUC	ACC	SEN	SPE
Unenhanced	Training	0.803 (0.733–0.872)	0.763 (0.761–0.765)	0.681 (0.571–0.791)	0.831 (0.751–0.912)
	Test	0.7 (0.57–0.83)	0.712 (0.706–0.718)	0.567 (0.389–0.744)	0.833 (0.712–0.955)
Enhanced	Training	0.811 (0.744–0.878)	0.75 (0.748–0.752)	0.667 (0.555–0.778)	0.819 (0.736–0.902)
	Test	0.708 (0.582–0.835)	0.712 (0.706–0.718)	0.533 (0.355–0.712)	0.861 (0.748–0.974)

AUC, area under the ROC curve; ACC, accuracy; SEN, sensitivity; SPE, specificity.

## Discussion

Radiomics by basic artificial intelligence is able to reach the pixel grayscale level, extract more information unseen by the naked eye, and obtain a variety of characteristics. Because the acquired information can be quantized, the objectivity of the results increases, and the accuracy of evaluating the change in disease is higher (8–10). Our study object was peripheral-type lung carcinoma, which manifests as a solid nodule on CT imaging. The methods of extracting radiomic features and establishing a prediction model were used to predict whether solid nodules had hilar and mediastinal lymph node metastases. Our study used ITK-SNAP software to extract all slices of primary tumors in unenhanced and enhanced CT images (15), and these images were input into AK software to extract first-order features and textural features (16). First, correlation analysis of features was performed between unenhanced and enhanced imaging, and our results indicated that the radiomic features of solid nodules had a high correlation between the two groups. Among them, shape features had the highest correlation (*r* = 0.98, median), and the result was in accordance with clinical observations. Second, based on the textural features of solid lung nodules on unenhanced and enhanced images, both of the established models could better predict hilar and mediastinal lymph node metastases, which suggests that the application of textural features promoted the potential of solid nodule lung cancer staging. Because our aim is studying biologic behaviour of solid nodes of lung cancer, the reliability of hilar and mediastinal lymph node of metastases is important. Just the CT and PET-CT manifestions of lymph node were observed but weren't analyzed furtherly.

In our study, a total of 107 extracted features were classified into 7 categories, including first-order features and second-order textural features. Correlation analysis of radiomics based on unenhanced and enhanced imaging has rarely been reported. In some references, the authors performed textural analysis of primary tumors for predicting lymph node metastasis in lung cancer and rectal carcinoma and established staging models, in which only unenhanced imaging in lung cancer (7) or enhanced imaging of the venous phase in rectal carcinoma (17) were used for textural analysis. There were no studies on the correlation or comparison between unenhanced and enhanced imaging nor were there any studies focused on establishing a model for unenhanced and enhanced imaging as our study did. Since the blood supply state greatly influences the biological behavior of tumors (18), we consider that it is important to investigate the radiomics features of unenhanced and enhanced imaging.

Our study evaluated the ability of radiomics to predict hilar and mediastinal lymph node metastases in solid nodule lung cancer and found that 5 features from 107 extracted features had predictive value in unenhanced and enhanced imaging. Two prediction models based on the multifactorial regression of unenhanced and enhanced imaging were established. The prediction efficiencies were as follows: in unenhanced imaging, the AUC was 0.803 in the training group, and the sensitivity and specificity were 0.681 and 0.831, respectively. The AUC was 0.7 in the test group, and the sensitivity and specificity were 0.567 and 0.833, respectively. In enhanced imaging, the AUC was 0.811 in the training group, and the sensitivity and specificity were 0.667 and 0.819, respectively. The AUC was 0.708 in the test group, and the sensitivity and specificity were 0.533 and 0.861, respectively. The above study results demonstrated that the prediction models had high prediction efficiencies and that the efficiencies were consistent in the training group and the test group, which revealed that our method was repeatable. Furthermore, the prediction efficiencies of the models in the training group and the test group between unenhanced and enhanced imaging were compared. The results were not significantly different (*p* > 0.05), showing that the two established models were able to replace each other.

Yifan Zhong et al. performed a study of hilar lymph node metastasis of lung cancer using a radiomic features + deep learning model (7), and their prediction model was compared with another 3 models (19–21). They found that the prediction efficiencies of several models exceeded 0.8. This result suggested that the radiomic method and established model were feasible and stable and supported our results. However, in the study by Yifan Zhong et al. the CT manifestations of lung cancer nodules included both solid nodules and ground-glass nodules (7). Because the probability of lymph node metastasis is very small in lung cancer with ground-glass nodules (22), it could subjectively increase the predictive efficiency and reduce the objectivity of the study result. In addition, Yifan Zhong et al. (7) did not list what textural features had predictive significance, which affects the intelligibility of the study method and results. It is worth mentioning that our study removed ground-glass nodules, clearly listed variables with predictive significance and stated that each type of textural feature was different in the unenhanced prediction model and the enhanced prediction model. In the unenhanced prediction model, the 5 features with predictive significance included 3 different GLRLM features, which express the similarity of the grayscale intensity values, the uncertainty/randomness of the grayscale distribution and the level of the heterogeneity of the texture patterns (22), respectively, 1 GLSZM feature and NGTDM-Strength, which express the distribution of the higher grayscale values and size of the region and the extent of the change, respectively (23). In the enhanced prediction model, the 5 features with predictive significance included 2 different GLDM features, which measure the proportion and directionality of the joint distribution of smaller size regions with lower grayscale values in the image, 2 different GLSZM features, which express a disordered state of the gray-degree value distribution, and 1 first-order feature, total energy, which expresses the energy features of the image (24). In our study, through the extracted textural features, the grayscale, uniformity, difference, and energy of the images were quantified at the pixel level, deeply revealing the tumor characteristics. These characteristics are related to the genetic structure, histological morphology and biological behavior of tumors (8).

Our study has several limitations. First, regarding the selection of patients, to ensure the reliability of the study, of 899 patients, only 218 patients with proven histopathology and complete clinical data were included, which led to many patients being excluded, and the observed data could have some bias. Second, most tumors included in this study were adenocarcinomas, and other pathological types, such as squamous cell carcinoma and adenosquamous carcinoma, were few and failed to be studied. Finally, only single-center data were used in our study. Confirmation by multicenter data should still be performed to ensure the generalization of our results.

In conclusion, 7 types of radiomic features were correlated between unenhanced and enhanced imaging. Both of the established models had high prediction efficiencies for predicting hilar and mediastinal lymph node metastases in lung cancer solid nodules, and the AUCs of both models exceeded 0.8. This result indicates that our study improved the accuracy of crucial staging in lung cancer. Additionally, it should be possible that either unenhanced imaging or enhanced imaging can be used to stage lung cancer solid nodules and achieve the aim of more simplified CT examinations.

## Data Availability

The raw data supporting the conclusions of this article will be made available by the authors, without undue reservation.
