# Supplementary figures and images for: Correlation analysis between unenhanced and enhanced CT radiomic features of lung cancers presenting as solid nodules and their efficacy for predicting hilar and mediastinal lymph node metastases
Source: Front Radiol. 2022 Oct 20;2:911179. doi: 10.3389/fradi.2022.911179 (PMC10365119; doi:10.3389/fradi.2022.911179)

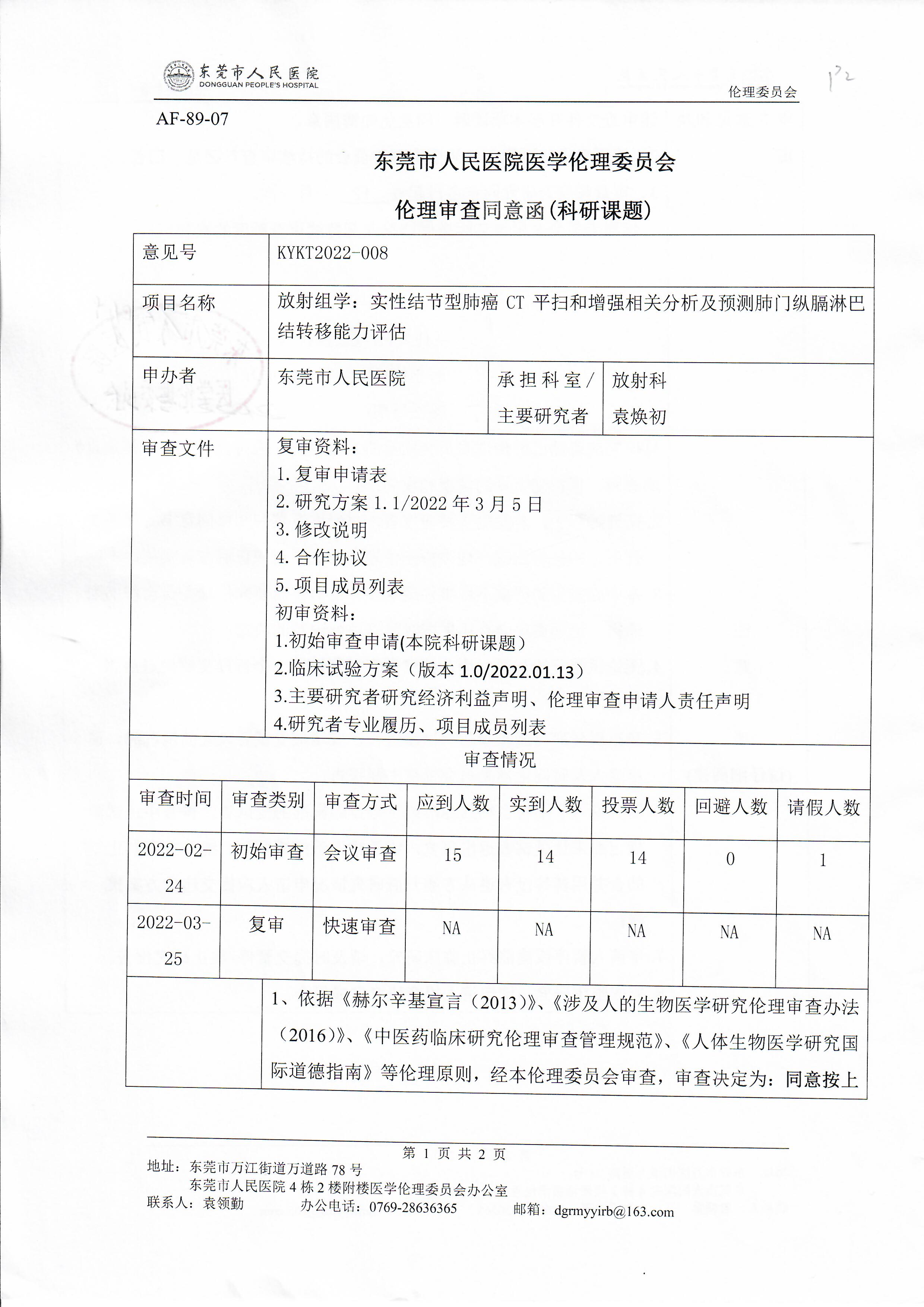

Supplement: Supplementary file 1 [file Image1.jpeg]

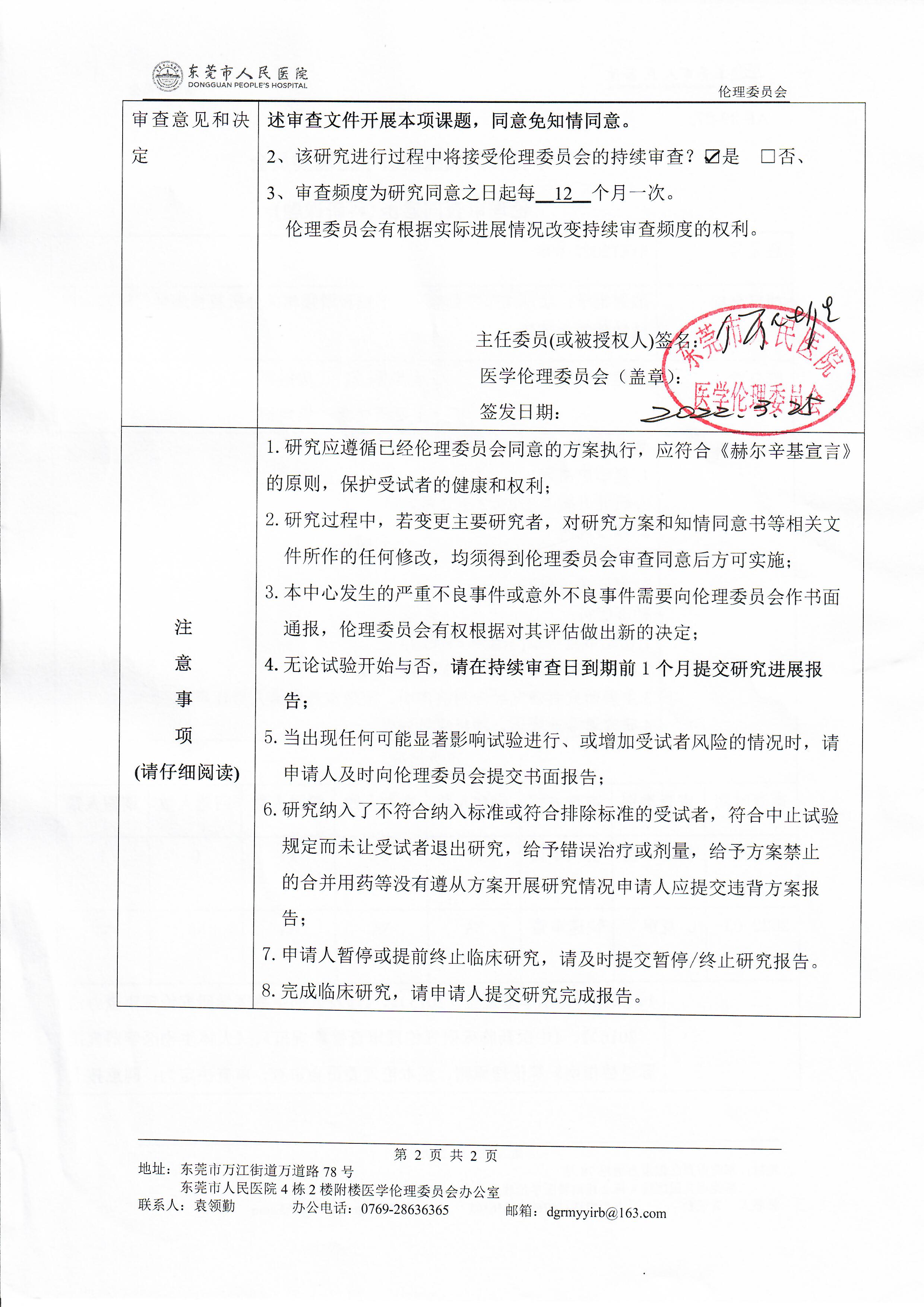

Supplement: Supplementary file 2 [file Image2.jpeg]

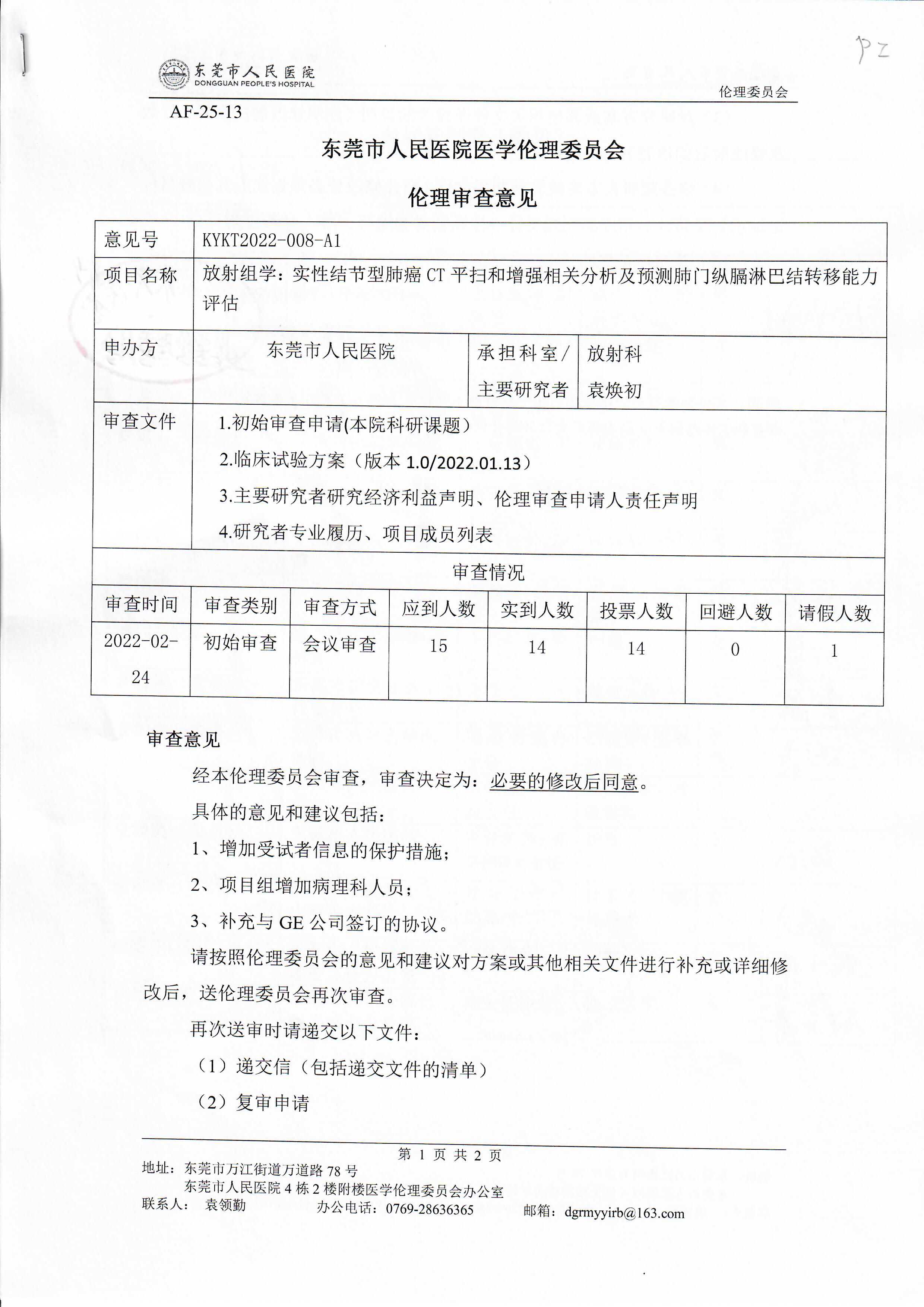

Supplement: Supplementary file 3 [file Image3.jpeg]

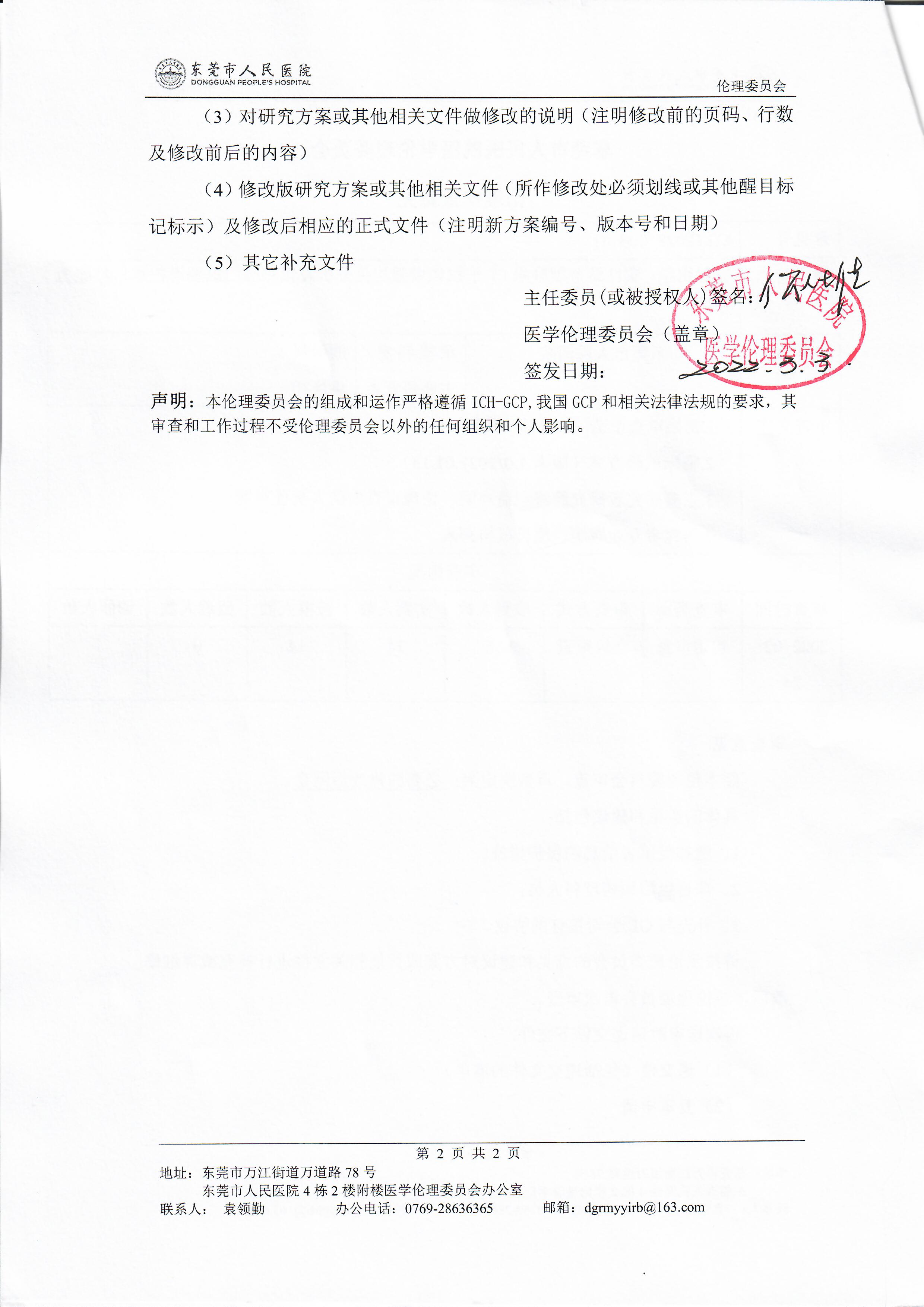

Supplement: Supplementary file 4 [file Image4.jpeg]

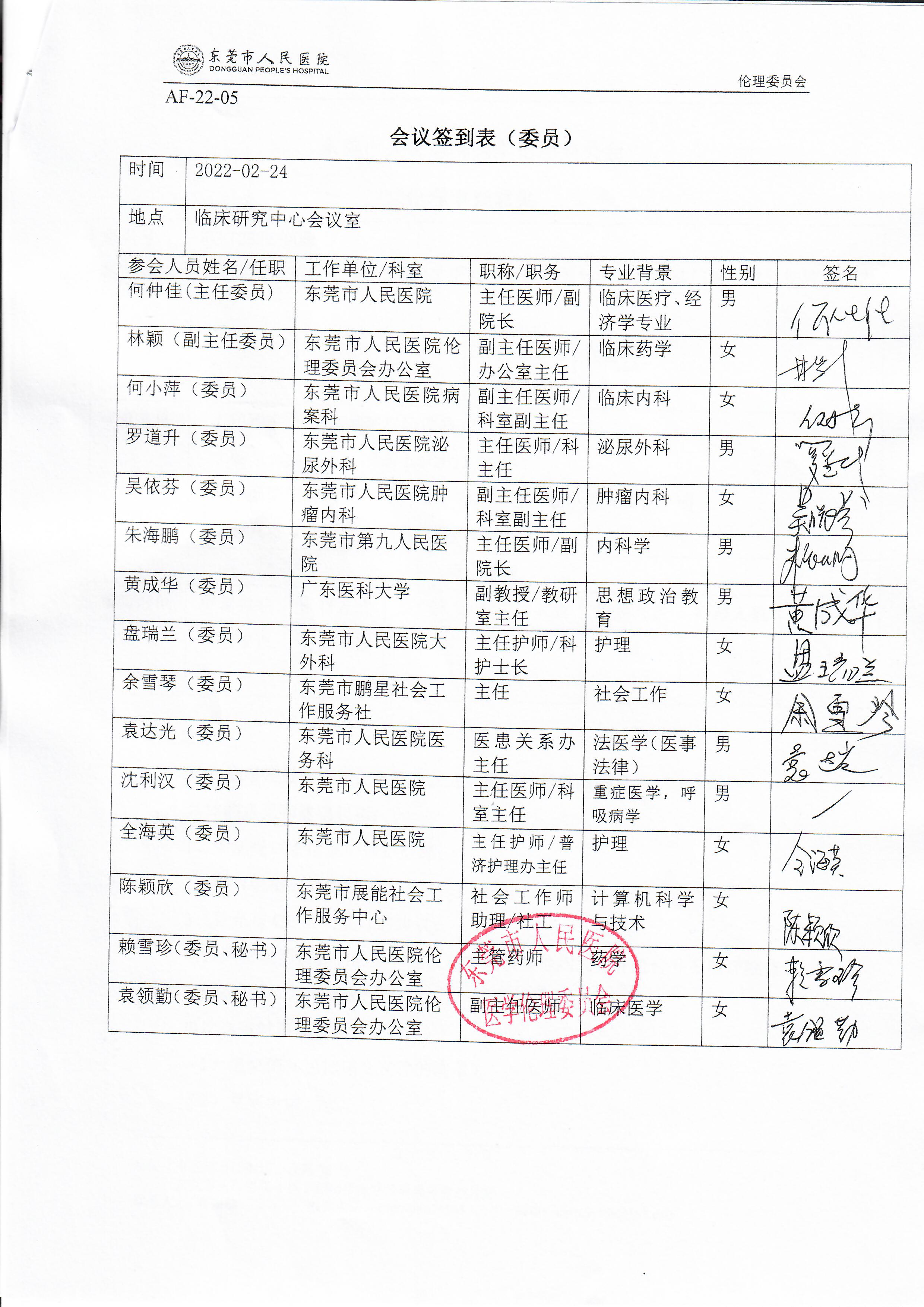

Supplement: Supplementary file 5 [file Image5.jpeg]
